# ‘Broken souls’ vs. ‘mad ax man’ – changes in the portrayal of depression and schizophrenia in the German media over 10 years

**DOI:** 10.1017/S204579602400043X

**Published:** 2024-09-18

**Authors:** M. Sittner, T. Rechenberg, S. Speerforck, M.C Angermeyer, G. Schomerus

**Affiliations:** 1 Department of Psychiatry and Psychotherapy, University of Leipzig Medical Centre, Leipzig, Germany; 2 Center for Public Mental Health, Gösing am Wagram, Gösing, Austria

**Keywords:** depression, media coverage, schizophrenia, stigma

## Abstract

**Aims:**

Population studies show the stigma of depression to diminish, while the stigma of schizophrenia increases. To find out whether this widening gap is reflected in the media portrayal of both disorders, this study compares the portrayal of depression and schizophrenia in German print media in 2010 vs. 2020.

**Methods:**

We conducted a qualitative content analysis using a mixed deductive-inductive approach to establish a category system. In total, we analyzed 854 articles with the summative approach by Mayring.

**Results:**

The study found a widening gap in the portrayal of schizophrenia and depression in German media between 2010 and 2020. Schizophrenia was depicted increasingly negative between 2010 and 2020, covering more negative stereotypes and focusing on its biological causes. Depression received increased attention and more neutral and professional coverage, with a greater emphasis on psychosocial causes and discussion of treatment options.

**Conclusions:**

By showing a widening gap the study highlights how media may shape public views on mental illnesses and reflects public attitudes at the same time. Media analyses from other nations have shown similar trends. This emphasizes the need for responsible reporting to combat stigma and promote understanding worldwide. Therefore, the authors recommend a balanced coverage that includes accurate professional information about all mental illnesses.

## Introduction

There appears to be a widening gap in the stigmatization in the general public between depression and schizophrenia (Schomerus *et al.*, 2021). This has been shown for the desire for social distance, pro-social actions and emotional reactions as an ongoing trend over the past 30 years (Schomerus *et al.*, 2022) and does not only occur in Germany but has also been observed in the USA (Pescosolido *et al.*, 2021). The widening gap has most recently become visible when comparing public attitudes towards continuum beliefs for the two disorders in 2011 and 2020 (Schomerus *et al.*, 2021). More people subscribed to continuum beliefs for depression between 2011 and 2020, while support for continuum beliefs in schizophrenia declined. Furthermore, the study showed a decrease in the perceived ‘otherness’ for depression, whereas it remained at a high level for schizophrenia (Schomerus *et al.*, 2021). Continuum beliefs are associated with less stigmatizing attitudes (Angermeyer *et al.*, 2015; Peter *et al.*, 2021), correlating with reduced fear and social distance, decreased perception of dangerousness and unpredictability, and stronger pro-social responses (Peter *et al.*, 2021). Moreover, according to Link & Phelan, the perception of ‘otherness’ is the centrepiece in the stigma process (Link and Phelan, 2020). It has been suggested that differences in media coverage could be one of the reasons for the widening gap in stigmatization (Rosset *et al.*, 2020; Schomerus *et al.*, 2021).

Many print media analyses from different countries worldwide have already revealed an often stereotypical portrayal of both disorders (e.g., Grandón *et al.*, 2022; Manago *et al.*, 2017; Nawková *et al.*, 2012; Ohlsson, 2018; Slopen *et al.*, 2007), depicting especially persons with schizophrenia as criminal or using ‘schizophrenic’ as a metaphor rather than a term for illness (e.g., Boke *et al.*, 2007; Chopra and Doody, 2007; Duckworth *et al.*, 2003; Hoffmann-Richter *et al.*, 1998; Rodrigues-Silva *et al.*, 2017). A content analysis conducted in 2018 on three German newspapers shed light on the divergence in the media coverage of depression and schizophrenia (Rosset *et al.*, 2020). First of all, depression received the highest media coverage among all psychiatric disorders, considerably surpassing schizophrenia (Rosset *et al.*, 2020). Second, there was a greater emphasis on medical information for depressive disorders, while schizophrenia was most often reported in relation to crime and criminality (Rosset *et al.*, 2020).

In the past, a correlation between print media coverage and attitudes was suspected, but so far, it has not been demonstrated in temporal conjunction with studies of public attitudes and to our knowledge, there has been no comparative study exclusively focusing on depression and schizophrenia in the German media.

This paper aims to address this gap of knowledge. Therefore, we conducted a qualitative content analysis of all articles mentioning depression or schizophrenia in three nationwide German newspapers for the years 2010 and 2020. We aimed to examine the evolving differences in media coverage of depression and schizophrenia between 2010 and 2020. This involved assessing the overall connotation of articles, the share in reporting, professional contributions, article sections and the quantity and type of reported stereotypes, causes and treatment options.

Given the results of the previous studies, we hypothesize that there will be a greater emphasis on depression in media coverage compared to schizophrenia. We expect a more qualitative and less stereotypical portrayal of depression, with a greater focus on providing medical information and discussion of treatment options. Furthermore, we anticipate that this difference will intensify between 2010 and 2020.

## Methods

### Selection of articles

We decided to analyze three newspapers that are considered influential in shaping public opinion, according to the criteria of Wilke (Wilke, 2009). These newspapers are *BILD Zeitung*, which had the highest daily sales in Germany in both 2010 and 2020 (Statista, 2021a, 2021b), *Süddeutsche Zeitung*, which is particularly popular among journalists (Wilke, 2009), and *Spiegel*, which was the most frequently cited newspaper overall in 2010 and 2020 (*‘Spiegel’: Nachrichtenmagazin bleibt meistzitiertes deutsches Medium,* 2010; Pfeffer, 2021). We received access to the archived material of the three newspapers via online archives. We included all regional issues, online articles and also extra issues like *Bild am Sonntag*, *BILD Plus* and *BILD Sport*. To filter suitable articles, we conducted a keyword search with the root words ‘depress’ and ‘schizophr’. To limit the number of articles, we narrowed the search period to the second quarter of 2010 and 2020. Our inclusion criteria were as follows: articles had to be in German, could be either printed or online, and search results could also include captions of pictures, headlines, announcements or advertisements. Additionally, we also accounted for articles where the terms ‘depress’ or ‘schizophr’ were used metaphorically, since we recognize that this figurative usage also shapes the readers’ understanding and view of the disorders. This way, a total number of 854 articles could be taken into account.

### Analysing the articles

In order to comprehensively analyze articles and extract valuable information, we employed two coding units. First, an Excel spreadsheet was created to capture details about the entire article, such as its overall connotation or the section in which it was published.

Second, we had a closer look at the text passages within the articles. These text passages were selected based on their inclusion of the root words ‘depress’ or ‘schizophr’ or their relevance to the meaning of these keywords and were treated as coding units. To evaluate these coding units, we used a qualitative content analysis, which is a systematic, rule-based, data-reducing procedure (Mayring and Fenzl, 2019). Specifically, the summative approach defined by Mayring suited our project best. Initially, a text passage was paraphrased by removing irrelevant text components or repetitive phrases that did not contribute to the core content. Subsequently, the paraphrased statements were generalized to a defined level of abstraction, ensuring that the original meaning was preserved in the new statement. Finally, redundant statements were eliminated, and the generalized information could now be assigned to a category based on predefined coding guidelines (Mayring and Fenzl, 2019). To assist in this process, we utilized MaxQDA (version 22.2.0) as a computer-assisted coding tool. Examples of our approach can be seen in Table 1.

**Table 1.** Examples of the summative approach in qualitative content analysis by Mayring

Text passage	Paraphrase	Generalization	Category
‘According to media reports, the single mother suffered from depression after her divorce.’^a^	After the divorce, the woman suffered from depression.	Divorce can be a trigger for depression.	Causes – psychosocial stress factors
The depressed man made a confession: he had stabbed the oldest of his three children.^b^	The depressed man stabbed his child.	People with depression may become violent.	Stereotypes – dangerousness
‘However, the trial is solely about whether the defendant, who suffers from schizophrenia, needs to be detained in a psychiatric hospital on a long-term basis.’^c^	The defendant, who suffers from schizophrenia, may be detained in a psychiatric hospital on a long-term basis.	People with schizophrenia may be detained in psychiatric care.	Therapy – detention

a Süddeutsche Zeitung (11.05.2010) Frau tötet Spielkameradin ihrer Tochter.

b Süddeutsche Zeitung (09.04.2010) Karlsruhe überprüft Urteil im Mordfall Büsra.

c Bildzeitung (10.06.2020) Prozess um tödliche Gartenzwerg-Attacke.

### Creating a category system

To establish our category system, we used a mixed deductive-inductive approach. The deductive aspect drew upon previous research studies that had analyzed print media. Consequently, main categories were formulated, encompassing aspects that apply to both schizophrenia and depression in a comparable manner (e.g., ‘treatment’ or ‘causes’). After reviewing 10% of the articles, these main categories were extended and supplemented with subcategories. While making sure the main categories stayed the same for schizophrenia and depression, subcategories could vary between the disorders, thereby indicating distinctions in news coverage for each disorder.

### Operationalization of the research questions

Based on previous studies of the portrayal of mental illness in print media (Angermeyer and Matschinger, 2004; Hayward and Bright, 1997; Hoffmann-Richter, 2000; Kroll *et al.*, 2003; Rottleb *et al.*, 2007; Schlier and Lincoln, 2014), we used the following operationalization:

*The overall connotation of the article:* After an initial reading of each article, we determined whether the connotation appeared appreciative, neutral or pejorative. For instance, if ‘depression’ was depicted within the context of crime or racism or if ‘schizophrenic’ was used metaphorically to describe something contradictory or demonic, it would be categorized as having a ‘pejorative connotation’. To assess the overall impression conveyed to readers, we also identified appreciative or pejorative text passages. We also took into account implicit evaluations, such as the use of terms like ‘psycho-clinic’ instead of ‘psychiatry’ or ‘psycho-prison’ instead of forensic psychiatry.

*The share in reporting for each disorder*: To measure the interest the media shows for both disorders, we compared the total number of articles about each one. Furthermore, we evaluated how many times the root words ‘depress’ and ‘schizophr’ appeared within one article (single or multiple references). We noted whether depression and schizophrenia were the main topics of the article, side topics, marginal notes or used metaphorically. Building from prior research (Hoffmann-Richter, 2000; Kroll *et al.*, 2003; Rottleb *et al.*, 2007), we designated articles as ‘main topic’ when the focus was on one of the disorders, and as ‘side topic’ when the focus was on another subject, but ‘schizophr’ or ‘depress’ were used to explain or influence a significant part of the article. When the root words were mentioned without significantly altering the article, we categorized it as a ‘marginal note’. Finally, metaphorical usage was considered as a transfer of meaning detached from the actual concept of the disorder.

*The* *number* *of* *times* *professionals* *get* *to* *speak* *in* *an* *article:* This category is intended to provide an indication of the professionally accurate portrayal of disorders in the media. The term ‘professionals’ refers to psychiatrists, psychologists, psychotherapists, alternative practitioners for psychotherapy or researchers in psychiatry-related areas. Non-specialist occupations and non-psychiatric researchers were categorized as ‘others’.

*The section an article is published in:* By assessing article sections, we determined how the media portrays disorders, whether it leans towards a scientific, political or cultural perspective, and how often those affected had an opportunity to voice their views. We assigned our own names for the sections, as the wording varied among the newspapers. The section ‘Medicine & Health’ included articles with specialized medical information, excluding the latest research findings, which were categorized under ‘Science, technology & research’. ‘Reports from affected people’ presented direct quotes from individuals, while journalistic discussions of affected individuals in the third person were categorized under ‘individual life stories’.

*The number and type of stereotypes the disorders are linked to*: Stereotypes are crucial in the process of stigmatization (Link and Phelan, 2020). We analyzed our articles based on the stereotypes initially defined by Hayward and Bright (1997) and subsequently used by Angermeyer and Matschinger (2004) and Schlier and Lincoln (2014) to be able to compare our results. We examined possible stereotypical portrayals in both coding units: text passages and whole articles. Coding units were labeled as ‘dangerousness’ when an affected person was portrayed as a threat to others, ‘unpredictability’ when a general inability to control oneself was implied, ‘personal negligence’ when a (partial) self-inflicted guilt of the affected person was suggested, and ‘incurability’ when depression or schizophrenia were presented as chronic disorders without effective cures. The only positive stereotype, ‘creativity and brilliancy’, referred to cases where affected individuals were depicted as exceptionally creative or ingenious.

*The number and type of reported causes of the disorders:* To capture the reported causes of the disorders, we built upon categories previously defined by other researchers (Holzinger *et al.*, 2001; Kroll *et al.*, 2003). These included ‘biological factors’ (e.g., heredity or neurotransmitter imbalances), ‘influences from socialization’ (e.g., violence in childhood, parental conflicts), ‘psychosocial stress factors’ (e.g., performance pressure, social isolation), ‘intrapsychic factors’ (e.g., unconscious conflicts) and other (e.g., fate, misfortune) (Kroll *et al.*, 2003). We inductively expanded these categories to include ‘combination of biological factors and environmental factors’ and ‘consumption of alcohol or drugs’. In 2020, we observed several phrases suggesting a pandemic-related origin of the disorders, leading us to add the subcategory ‘COVID-related stress’ within the ‘psychosocial stress factors’ category. This subcategory included persistent stress, anxiety, social isolation and loneliness triggered by the COVID-19 pandemic and associated mobility restrictions.

*The number and type of reported treatment options for the* *disorders*: To evaluate the reported treatments we distinguished between established psychiatric methods (e.g., psychotherapy, psychopharmacotherapy or psychosocial rehabilitation) and other methods (e.g., music, arts and sports therapy, as well as self-healing, retribution, telephone counselling). In the top-level category ‘treatment’, we also included the subcategories ‘start, course and outcome of treatment’ (e.g., passages discussing treatment duration), ‘detention’ (related to a crime) and ‘prevention’ of the disorders.

### Proof of reliability

For reliability testing, a sample of 10% of the articles was randomly selected and re-coded by TR. Consistency was determined by considering it a match when both coding persons assigned the same code an equal number of times in an article. After discussing non-agreements, some categories were renamed, defined more precisely and distinguished better from other categories. Reliability for the coding unit ‘article’ was evaluated using kappa and interpreted following Landis and Koch (1977), resulting in kappa from 0.644 (substantial) to 0.959 (almost perfect, for details, see table S1). For the coding unit ‘text passage’, an interrater agreement of 80.19% was calculated with the assistance of MaxQDA.

## Results

We found that articles on schizophrenia generally leave a more negative impression than those on depression. In 2020, the *overall* *connotation* of these articles is even more pejorative compared to 2010 and additionally, we found more derogatory text passages, which degrade sufferers for example as ‘mad’ (DER SPIEGEL(03.04.2020) Zum Gefechtstraining in die Slowakei) or ‘being nuts’. (BILD Deutschland (25.06.2020) Irrer Axt-Schläger greift Rentnerin an). In contrast to this, the reporting on depression is perceived as predominantly neutral. Between 2010 and 2020, the number of articles with negative connotation and the number of text passages categorized as pejorative decreases (for detailed data, see Table 2). Nevertheless, also in reports about depression we find attention-grabbing headlines, miscalling psychiatrist institutions for instance as ‘island of broken souls’ (SPIEGEL Wissen (03.05.2010) Insel der kaputten Seelen).

**Table 2.** Results of the categories with coding unit ‘article’

	Depression 2010 percent (*n*)	Depression 2020 percent (*n*)	Schizophrenia 2010 percent (*n*)	Schizophrenia 2020 percent (*n*)
**Total number of articles**	100 (326)	100 (432)	100 (56)	100 (40)
**Overall connotation of the article**				
Pejorative	44.2 (144)	18.3 (79)	66.1 (37)	77.5 (31)
Neutral	55.2 (180)	79.9 (345)	30.4 (17)	20.0 (8)
Appreciative	0.6 (2)	1.9 (8)	3.6 (2)	2.5 (1)
**Frequency of reporting**				
‘depress’/‘schizophr’ used as				
single reference	82,5 (269)	76.6 (331)	85.7 (48)	87.5 (35)
multiple references	17.5 (57)	23.4 (101)	14.3 (8)	12.5 (5)
main topic	7.7 (25)	4.9 (21)	5.4 (3)	0 (0)
side topic	34.7 (113)	38.4 (166)	39.3 (22)	50 (20)
marginal note	23.0 (75)	27.6 (119)	23.2 (13)	20 (8)
metaphor	34.7 (113)	29.2 (126)	32.1 (18)	30 (12)
**Professionalism in reporting**				
‘depress’/‘schizophr’ used by				
professionals	12.0 (39)	23.8 (103)	19.6 (11)	12.5 (5)
others	88.0 (287)	76.2 (329)	80.4 (45)	87.5 (35)
**Sections**				
Medicine & health	8.9 (29)	14.8 (64)	1.8 (1)	7.5 (3)
Science, technology & research	5.5 (18)	2.8 (12)	21.4 (12)	2.5 (1)
Economy & finances	12.0 (39)	19.4 (84)	3.6 (2)	2.5 (1)
Culture & arts	22.1 (72)	15.3 (66)	23.2 (13)	27.5 (11)
Sports	9.2 (30)	1.2 (5)	10.7 (6)	2.5 (1)
Politics	8.0 (26)	6.0 (26)	3.6 (2)	15 (6)
Reports from affected people	4.6 (15)	5.1 (22)	1.8 (1)	2.5 (1)
Individual life stories	19.0 (62)	8.6 (37)	25.0 (14)	37.5 (15)
Society	10.7 (35)	26.9 (116)	8.9 (5)	2.5 (1)
**Stereotypical portrayal**				
Dangerousness	8.3 (27)	5.3 (23)	26.8 (15)	40.0 (16)
Unpredictability	3.1 (10)	1.2 (5)	12.5 (7)	15.0 (6)
Creativity & brilliancy	1.2 (4)	0.5 (2)	8.9 (5)	2.5 (1)
Incurability	0.3 (1)	0.2 (1)	7.1 (4)	0.0 (0)
Personal negligence	0.0 (0)	0.2 (1)	0 (0)	0.0 (0)

*The share in reporting* for depression is higher than for schizophrenia in both years. Between 2010 and 2020 the priority in news coverage increased for depression, while it decreased for schizophrenia. In fact, the number of articles about depression is more than ten times higher in 2020 than about schizophrenia, while it was only about six times higher in 2010. But not only does the reader get more information about depression, the information is also given more often by *professionals.* Whereas in 2010 more professionals had their say in the news coverage on schizophrenia than on depression, in 2020 the opposite presents.

Upon reviewing the *sections* in which articles were published, more noticeable changes between the two compared years become apparent. News on schizophrenia remain mainly in the department ‘individual life stories’, in which – for schizophrenia – only criminal cases are reported. Here, the number of articles in this section rises in 2020. The articles captivate readers with striking headlines, unveiling the most bizarre and brutal crimes committed by individuals with schizophrenia, as later revealed within the pages. *BILD Zeitung* for example published a report titled ‘mad ax man attacks pensioner’^2^or *Süddeutsche Zeitung* informs about an ‘attack with potato peeler’ (Süddeutsche Zeitung München (17.06.2010) Attacke mit dem Kartoffelschäler) planned by a person with schizophrenia. In contrast, coverage of depression in the same category of individual life stories is comparatively lower overall and declined further in 2020 compared to 2010. Here, alongside a few criminal cases, the section holds various reports on people who overcame the disorder or prevention tips from affected celebrities. In 2020, news reports on depression are most commonly part of the section ‘society’. For both disorders, there are few reports in the ‘medicine & health’ section.

**Figure 1.**
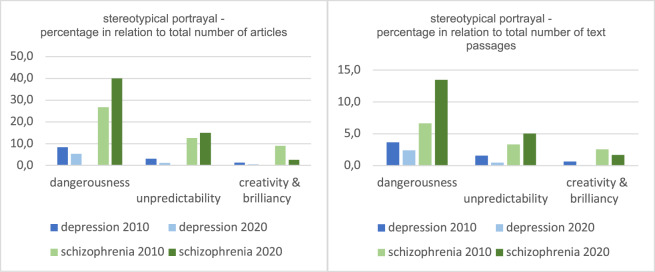
Development of the stereotypical portrayal of schizophrenia and depression between 2010 and 2020 (main results).

In exploring *the stereotypical portrayal* in the media coverage, we observed similar trends in both coding units, articles and text passages (see Tables 2 and 3 and Fig. 1). Both disorders are most frequently associated with the stereotype of ‘dangerousness’, with individuals with schizophrenia being portrayed as significantly more dangerous overall than those with depression, and this portrayal increased further in 2020 compared to 2010. The same applies to the stereotype ‘unpredictability’, which is also often used. This results in 40% of all articles reporting on schizophrenia in 2020 describing sufferers as dangerous and 15% describing them as unpredictable. Taking into consideration that in another 30% of all articles ‘schizophr’ is only used metaphorically, this leaves very little room for informative content. Furthermore, the only positively connoted stereotype of ‘creativity & brilliancy’ is used more often for schizophrenia than for depression. However, this positive representation decreases from 8.9% to 2.5% of all articles between 2010 and 2020.

**Table 3.** Results of the categories with coding unit ‘text passages’

	Depression 2010 percent (*n*)	Depression 2020 percent (*n*)	Schizophrenia 2010 percent (*n*)	Schizophrenia 2020 percent (*n*)
**Total number of text passages**	100 (764)	100 (1081)	100 (271)	100 (119)
**Text passages with evaluative tone**				
Appreciative	0.9 (7)	0.3 (3)	0.0 (0)	0.0 (0)
Pejorative	8.5 (65)	3.5 (38)	7.7 (21)	12.6 (15)
**Stereotypical portrayal**				
Dangerousness	3.7 (28)	2.4 (26)	6.6 (18)	13.4 (16)
Unpredictability	1.6 (12)	0.5 (5)	3.3 (9)	5.0 (6)
Creativity & brilliancy	0.7 (5)	0.1 (1)	2.6 (7)	1.7 (2)
Incurability	0.1 (1)	0.1 (1)	1.5 (4)	0.0 (0)
Personal negligence	0.0 (0)	0.1 (1)	0.0 (0)	0.0 (0)
**Causes**				
Biological factors	1.0 (8)	0.7 (8)	10.3 (28)	1.7 (2)
Combination of biological factors & environmental factors	0.0 (0)	0.1 (1)	3.7 (10)	0.0 (0)
Influences from socialization	0.9 (7)	1.9 (20)	0.4 (1)	0.8 (1)
Psychosocial stress factors	9.2 (70)	5.7 (62)	2.2 (6)	0.8 (1)
COVID-related stress	0.0 (0)	9.3 (101)	0.0 (0)	0.0 (0)
Consumption of alcohol or drugs	0.9 (7)	0.3 (3)	2.6 (7)	1.7 (2)
Intrapsychic factors	0.3 (2)	0.1 (1)	0.0 (0)	0.0 (0)
Other	2.4 (18)	2.9 (31)	0.0 (0)	0.8 (1)
Total	14.7 (118)	21.0 (227)	19.2 (52)	5.9 (7)
**Treatment**				
Established psychiatric methods	7.5 (57)	9.4 (102)	8.1 (22)	2.5 (3)
Other methods	2.0 (15)	5.8 (63)	2.2 (6)	0.8 (1)
Start, course and outcome of treatment	1.4 (11)	3.5 (38)	1.5 (7)	1.7 (2)
detention	0.4 (3)	0.1 (1)	2.6 (4)	10.1 (12)
prevention	0.0 (0)	1.9 (21)	0.0 (0)	0.0 (0)
Total	11.3 (86)	20.8 (225)	14.4 (39)	15.1 (18)

Shifting our focus to the reported *causes* of depression and schizophrenia, we also found notable variations between the compared years. In 2010, there are more text passages about the causes of schizophrenia than those of depression. In 2020, the opposite occurs and causes for depression take the lead, while the percentage of text passages on causes for schizophrenia decline drastically from 19.2% to 5.9%. But not only the number but also the type of reported causes differs: For schizophrenia, biological factors are predominantly mentioned in both years. In 2010, a combination of biological factors and environmental factors is also frequently brought up, which is in line with the current professional opinion. In 2020, on the other hand, next to biological factors, the consumption of alcohol or drugs is presented as a prominent factor contributing to schizophrenia. In articles addressing depression on the other hand, psychosocial stress factors are seen as the main cause for the disorder, increasing in-between 2010 and 2020 from 9.2% to 15% of all text passages. In 2020, the stress due to the COVID pandemic and the associated limitations thereby play the most important role, covering a noteworthy 9.3% of all analyzed text passages. In contrast to that, not a single article mentions schizophrenia in relation to COVID.

The category of *‘treatment’* aligns with the previously described trend. The proportion of text passages about treatment options for depression increases from 11.3% to 20.8%, while the numbers stay approximately the same for schizophrenia. Notably, there is a clear emphasis on the detention of individuals with schizophrenia in psychiatric institutions, particularly in 2020 compared to 2010. In fact, media reports on detention is four times more than about established psychiatric treatment in 2020. In the reporting on depression, established psychiatric procedures such as medications or psychotherapy are prominent in both years. Lastly, discussions on prevention are exclusively found in the news coverage on depression, and only in the year 2020.

## Discussion

Our qualitative content analysis of newspaper articles showed a widening gap between both disorders in all examined research questions. Depression got covered more often and there is a more *professional* picture drawn, while schizophrenia got covered less and with less reference to professional care between 2010 and 2020. The trend towards reporting more about depression, which Kroll *et al.* (2003) already noted in German print media between 1990 and 2000, thus seems to be continuing.

While for both disorders we found very little reports in sections dedicated to medicine and health, there is a big interest in crimes, especially when committed by people with schizophrenia. In 2010, there were a number of articles about new research on schizophrenia and its causes, but this scientific interest was nearly completely non-existent in 2020. For depression, in 2020 we found most articles in the section ‘society’, which we see strongly connected to the COVID-19 pandemic. The worsening of a pre-existing depression seems to be the prime example to show negative side effects of establishing too many restrictive rules, while there is not a single mention of schizophrenia in this context. Also, the media begin to talk about the prevention of depression – but never of schizophrenia.

In 2020, individuals with symptoms of schizophrenia are portrayed as more dangerous and unpredictable. The frequent reproduction of the stereotype ‘dangerousness’ corresponds to the research results of Schlier and Lincoln (2014) from an analysis of German print media in 2011. On the other hand, every single *stereotype* is used less frequently for individuals with depression in 2020. Also, the German newspapers draw a picture of a close relationship between ‘genius’ and ‘madness’ only for schizophrenia (Lombroso, 1864), but vanishingly little for depression. But the use of this one positive stereotype of creativity also decreases for schizophrenia between 2010 and 2020. Altogether, the reader is prone to an understanding of people with schizophrenia as a threat to society. *Stereotypes* are a key component of theoretical models of stigma, as they are believed to instigate adverse emotional reactions and discriminatory behaviour (Schomerus *et al.*, 2012). Therefore, it is even more concerning that the widening gap also enlarges for the stereotypical portrayal of depression and schizophrenia in the German media.

The media views biological factors as the predominant *cause* for schizophrenia in both years. This corresponds with the stereotypical portrayal of persons with schizophrenia as dangerous or unpredictable, even more so since several studies have linked biogenetic causal theories and the perception of dangerousness and unpredictability (Angermeyer *et al.*, 2022; Read *et al.*, 2006; Read and Law, 1999). Also, we assume that the increased mention of drugs and alcohol as the cause of schizophrenia in 2020 contributes to the increasingly negative image about the disorder.

The media’s emphasis on psychosocial factors as the primary cause of depression aligns with previous analyses of German print media (Kroll *et al.*, 2003) and the beliefs held by patients themselves (Holzinger *et al.*, 2001). There exists substantial evidence of reduced discrimination, fear and distance if psychiatric symptoms are viewed as understandable responses to life events (Longdon and Read, 1864; Walker and Read, 2002). Therefore, increased media coverage on psychosocial causes for depression might be part of the explanation why we see less stigmatization on depression over the past decades in Germany and the USA (Pescosolido *et al.*, 2021; Schomerus *et al.*, 2022).

As highlighted in the study introduction, various international cross-sectional studies have reported similar findings regarding the depiction of stereotypes or metaphorical usage, aligning with our study A longitudinal study conducted in England also revealed an improvement in reporting on depression over time, but negative portrayals persisted for schizophrenia in a comparison between 1992 and 2008 (Goulden *et al.*, 2011). More recently, a media analysis of English newspapers found a widening gap in stigmatization of depression and schizophrenia similarly to our study between 2008 and 2019 (Hildersley *et al.*, 2020). Given these observations, there is a strong indication that the findings of our study may demonstrate broad applicability across other nations.

### Limitations

Although this study has substantial findings, it is essential to consider the broader context of media consumption in Germany. Given that television and radio remain the most widely used media (Leibiger and Giersberg, 2020), newspapers may not be the most important influence on the public’s opinion about psychiatric disorders. In fact, newspapers and magazines, including online articles, account for only 3.3% of daily media consumption (Leibiger and Giersberg, 2020), and their reach continues to slowly decline (Adler *et al.*, 2022). Consequently, further studies should prioritize moving image and audio media, with particular attention to emerging social media platforms like Instagram and TikTok, since their frequency of usage has been increasing especially among younger people (Adler *et al.*, 2022).

Another limitation of this study may be the late reliability check. Ideally, a second intercoder could have conducted an analysis of a subset of articles during the category system’s development to detect potential weaknesses earlier in the project. Conducting the reliability check after completing the coding of all articles, we later realized that certain coding instructions lacked the requisite specificity. Consequently, we made minor adjustments to our category system towards the project’s conclusion.

### Conclusion

This research was conducted to understand the interrelations between media portrayal and the widening gap in public stigmatization between depression and schizophrenia observed between 2010 and 2020 (Schomerus *et al.*, 2021). We found a widening gap in all investigated categories. The study’s main findings reveal an intensifying negative and less professional portrayal of schizophrenia as well as an increasing prominence of depression, its treatment and prevention in media coverage. Although these findings are in line with the changes in continuum beliefs, we must consider that the portrayal of schizophrenia and depression in German media can be both a result and a cause of a stigmatizing image of society regarding these conditions.

Based on these findings, we recommend a balanced coverage including accurate professional information about the causes, treatment and prevention of *all* mental illnesses worldwide, but in particular with regard to schizophrenia. One could argue, it is not the media’s responsibility to educate about disorders, but the primary emphasis should not centre on the exploitation of individual biographies or the pursuit of unusual or sensational narrative. Journalists should be mindful of the spread of stigma in the media and its effects. Before reporting on a person with schizophrenia, media professionals should carefully consider whether discussing the illness is essential for the report and ensure they respect the privacy of those affected (Aktionsbündnis Seelische Gesundheit 2022). Sensitized reporting includes informative headlines, medical terminology and avoiding generalizations (Aktionsbündnis Seelische Gesundheit 2022). Also, the media should let affected persons have their say more often instead of writing about them from an evaluative outside perspective and reduce the proportion of the metaphorical use of ‘schizophrenic’, as this often reinforces the image of a split personality (Schomerus *et al.*, 2021). Our findings may help to develop issue-specific anti-stigma campaigns worldwide. This way, we may be able to combat stigma and promote understanding.

## Supporting information

Sittner et al. supplementary material Sittner et al. supplementary material

## Data Availability

The data that lead to the findings of this study are available on request from Madeleine Sittner.

## Supplementary material

The supplementary material for this article can be found at https://doi.org/10.1017/S204579602400043X.
