# Supplementary material for: ‘Broken souls’ vs. ‘mad ax man’ – changes in the portrayal of depression and schizophrenia in the German media over 10 years
Source: Epidemiol Psychiatr Sci. 2024 Sep 18;33:e37. doi: 10.1017/S204579602400043X (PMC11450423; doi:10.1017/S204579602400043X)
Supplement: Sittner et al. supplementary material [file S204579602400043Xsup001.pdf]

"Broken souls" vs. "mad ax man" - Changes in the portrayal of depression and schizophrenia in the German media over 10 years

M. Sittner, T. Rechenberg, S. Speerforck, MC. Angermeyer, G. Schomerus
corresponding author: Madeleine Sittner (Madeleine.Sittner@gmail.com)
Epidemiology and Psychiatric Sciences

Supplementary Material

Table S1: Results of reliability testing for coding unit "article"

category	kappa	SD	95% CI	evaluation
overall connotation of the article	0,644	0,08	0,487-0,800	substantial
frequency of reporting<br>main / side topic, marginal note or metaphor	0,656	0,062	0,534-0,779	substantial
single/multiple reference	0,959	0,041	0,879-1,000	almost perfect
sections	0,756	0,047	0,691-0,875	substantial
professionalism in reporting	0,851	0,084	0,686-1,000	almost perfect
stereotypical portrayal	0,69	0,079	0,535-0,846	substantial
